# Hemostatic effect of tourniquet combined with tranexamic acid in total knee arthroplasty: a network meta-analysis

**DOI:** 10.1186/s13018-020-02010-z

**Published:** 2020-11-12

**Authors:** Yimin Zhang, Bao Lang, Guifeng Zhao, Fengming Wang

**Affiliations:** 1 Joint Surgery Department, Weifang People’s Hospital, Weifang, 261000 People’s Republic of China; 2 Anesthesiology Department, Weifang People’s Hospital, Weifang, 261000 People’s Republic of China; 3 Medical Department, Weifang People’s Hospital, No. 151, Guangwen Road, Kuiwen District, Weifang, 261000 Shandong Province People’s Republic of China; 4 Orthopaedics Department, People’s Hospital of Xiashan Ecological and Economic Development Zone, No. 1, Xiashou Road, Xiashan District, Weifang, 261325 Shandong Province People’s Republic of China

**Keywords:** Total knee replacement, Tourniquets, Tranexamic acid, Hemostasis, Randomized controlled trial

## Abstract

**Background:**

There are various techniques to reduce blood loss in total knee arthroplasty (TKA), including the use of a tourniquet and tranexamic acid (TXA). In this study, we studied the combined effect of TXA with a tourniquet on blood loss in the setting of primary TKA.

**Methods:**

Randomized controlled trials (RCTs) of nine treatment methods were included (placebo, intravenous [i.v.] TXA, topical TXA, i.v.-combined topical TXA, oral TXA, placebo + tourniquet, i.v. TXA +tourniquet, topical TXA + tourniquet, and i.v.-combined topical TXA + tourniquet). The patients were divided into eight groups according to the different treatment strategies, with 30 cases per group. The differences in the total blood volume, the number of patients transfused, the hemoglobin before and after the operation, and complications after the operation were compared.

**Results:**

Totally 15 RCTs meeting our inclusion criteria were collected in this study. Compared with the placebo + tourniquet group, the i.v. TXA + tourniquet group displayed lower hemoglobin reduction value, pulmonary embolism (PE) incidence, total blood loss, and blood transfusion risk; the topical TXA + tourniquet group showed reduced PE incidence, total blood loss, and blood transfusion risk, and the i.v.-combined topical TXA and i.v.-combined topical TXA + tourniquet groups showed decreased total blood loss and lower blood transfusion risk. Retrospective clinical study results also demonstrated that the efficacy of i.v.-combined topical TXA was the best.

**Conclusions:**

Our meta-analysis indicates that i.v.-combined topical TXA provides a low total blood loss without increasing the blood transfusion risk in patients undergoing total knee replacement surgery.

## Background

Total knee arthroplasty (TKA) remains one of the most successful orthopedic operations to relieve pain and improve function in patients with arthritis [1]. The prevalence of TKA has increased dramatically due to the aging of the population and a rise in per capita utilization [2]. TKA leads to excessive perioperative blood loss, which can result in anemia and blood transfusions [3]. Indeed, allogenic transfusion is the standard method for managing acute blood loss following TKA [4]. However, the use of allogeneic transfusion can lead to undesirable adverse events such as infection, immunologic reaction, and even myocardial infarction [5]. Many blood preservation strategies have been proposed to reduce the operation-related blood loss and minimize the risk of postoperative blood transfusion, such as the use of the antifibrinolytic medication tranexamic acid (TXA) and application of a tourniquet [6, 7].

The application of tourniquet in TKA can improve the operative field of vision, reduce intraoperative blood loss, and improve the quality of cementation by providing a relatively bloodless operation site [8]. Nonetheless, the clinical role of the tourniquet in TKA remains a controversial matter. Ajnin et al. have proposed that TKA without tourniquet could reduce post-operative pain, swelling, and occult blood loss, including blood oozing into adjacent soft tissues and knee joints, thus improving the range of activity and enabling the entry of patients in rehabilitation training, while reducing the length of hospital stay [9]. Indeed, tourniquet use after TKA can provoke various complications, including a higher rate of deep vein thrombosis (DVT), wound infections, and nerve damage [10]. A recent study has shown that tourniquet application can decrease muscle strength and reduce knee range of motion (ROM), thus interfering with functional recovery after TKA [11].

Other research shows that tranexamic acid (TXA) treatment in conjunction with a tourniquet could compensate for the disadvantages of tourniquet use [7]. TXA, as an antifibrinolytic agent, is a synthetic lysine derivative, which can reduce blood loss by reversibly competing for the binding site of lysine on plasminogen to fibrin [12]. Accumulating evidence has also confirmed the benefits of TXA in decreasing blood loss and the transfusion rate in TKA [13]. In recent decades, the efficacies of intravenous, oral, and topical use of TXA has been compared in numerous studies, without showing distinct benefits from one route of administration [14]. A meta-analysis study compared the efficacy of topical intra-articular administration TXA versus placebo in TKA, which showed similar efficacies in reducing blood loss, hemoglobin reduction and the requirement for blood transfusion, without increasing adverse reactions [15]. Moreover, Lin at al. have confirmed that i.v.-combined topical with TXA could decrease the total blood loss and subsequent need for transfusion without increasing the occurrence of DVT in comparison to the topical, intravenous TXA alone [16]. The literature is largely unclear regarding the best-practice use of the tourniquet and TXA in the context of TKA surgery. Consequently, we made a meta-analysis of the nine treatment methods, aiming to determine if the use of a tourniquet in conjunction with TXA would improve total blood loss, transfusion rate, and complication rates.

## Material and methods

### Data sources

Relevant references were retrieved manually using the PubMed, EMBASE, Cochrane Library, and other English databases. The search range extended from the establishment to December 2019, with the use of combinations of keywords, i.e., total knee replacement, tourniquet, tranexamic acid, total knee arthroplasty, and randomized controlled trial (RCT).

### Inclusion and exclusion criteria

Inclusion criteria: (1) study type: randomized controlled study; (2) intervention measures: placebo, intravenous (i.v.) TXA, topical TXA, i.v.-combined topical TXA, oral TXA, placebo + tourniquet, i.v. TXA + tourniquet, and topical TXA + tourniquets; (3) study subjects: patients (over 45 years old) who suffered from osteoarthritis or rheumatoid arthritis have undergone TKA for the first time; (4) results, including blood loss or transfusion risk. Exclusion criteria: (1) patients had previously received knee surgery; (2) patients who received bilateral knee arthroplasty; (3) patients with a history of deep vein thrombosis (DVT) and pulmonary embolism (PE); (4) patients with osteoporosis; (5) patients with severe anemia (Hb <60 g/L), coagulation dysfunction and peripheral vascular diseases; (6) non-randomized controlled study design; (7) republished literature, (8) conference reports, systematic reviews or abstract article; and (9) non-English literature.

### Data extraction and quality evaluation

Using a unified data collection table, two researchers independently extract the data included in the literature search. If a dispute emerged in the data extraction process, the dispute would be solved by multiple researchers. Randomized controlled trials were evaluated by more than two researchers according to the Cochrane risk bias assessment tool [17]. The tool included six fields: random assignment, assignment concealment, blinding, loss of outcome data, selection of outcome reports, and other biases. The assessment included assigning a judgment of “yes,” “no,” or “unclear” for each domain to designate a low, high, or unclear risk of bias, respectively. If one or no domains were deemed “unclear” or “no”, the study was classified as having a low risk of bias. If two or three domains were deemed “unclear” or “no,” the study was classified as having a moderate risk of bias, whereas the study was classified as having a high bias risk if four or more domains were deemed “unclear” or “no” [18]. Quality assessment and investigation of publication bias were carried out using Review Manager 5 (RevMan 5.2.3, Cochrane Collaboration, Oxford, UK).

### Follow-up retrospective cohort study protocol

#### Patient data

The retrospective case-control study was carried out in the People’s Hospital of Xiashan Ecological and Economic Development Zone. The data of TKA patients collected prospectively from December 2015 to December 2019 were reviewed. According to different surgical treatment measures, patients were divided into eight groups (i.v. TXA, topical TXA, i.v.-combined topical TXA, oral TXA, Placebo + tourniquet, i.v. TXA + tourniquets, Topical TXA + tourniquet, and i.v.-combined topical TXA + tourniquet). For each group, 32 cases (61 males and 95 females; ranging in age from 45 to 75 years with a mean age of 66.01 ± 4.98) years) meeting the inclusion criteria were randomly selected for follow-up analysis to compare their total blood loss, risk of blood transfusion, changed hemoglobin values, and postoperative complications.

Inclusion criteria in the retrospective study: (1) patients were over 45 years old when undergoing surgery; (2) patients had osteoarthritis; (3) patients received TKA for the first time; (4) and patients were treated with TXA or tourniquet. Exclusion criteria: (1) patients had previously received knee surgery; (2) patients with a history of DVT and PE; (3) patients with osteoporosis; (4) patients with severe anemia, coagulation dysfunction, and peripheral vascular diseases (Hb <60 g/L). This study was approved by the internal review committee for orthopaedic research as an observational human study. All study data and information collected from patient files are confidential and are subject to the Declaration of Helsinki.

#### Operative method

All operations were performed by the same orthopedic operation group under similar anesthesia and operation protocols. No drainage tube was placed during the operation. In the first group, patients received an i.v. injection of 1.0 g TXA 10 min before incision and without tourniquet during the operation (i.v. TXA group). In the second group, after the closure of the joint capsule, patients were injected into the joint with 1 g/20 mL normal saline TXA once without tourniquet during the operation (Topical TXA group). In the third group, patients were intravenously injected with 1.0 g TXA at 10 min before surgical incision and 1 g/20 mL normal saline TXA was injected into the joint after closure of the joint capsule, and without tourniquet during the operation (i.v.-combined topical TXA group). In the fourth group, 2 g (4 tablets, 500 mg each) TXA was administered orally 2 h before the operation, and 1 g was repeated 6 h and 12 h after the operation, respectively, without tourniquet during the operation (Oral TXA group). In the fifth group, patients were intravenously injected with 10 mg/kg placebo before and 3 h after the release of the tourniquet, without treatment with TXA. Tourniquet was used throughout the operation. The tourniquet was released after the incision was sutured and pressure bandaged (placebo + tourniquet group). In the sixth group, patients were intravenously injected with 1.0 g TXA 10 min before incision, in conjunction with the tourniquet used throughout the operation. The tourniquet was released after the incision was sutured and pressure bandaged (i.v. TXA + tourniquets group). In the seventh group, after the closure of the joint capsule, patients were injected with 1 g/20 mL normal saline TXA once in the joint with tourniquet used throughout the operation. The tourniquet was released after the incision was sutured and pressure bandaged (Topical TXA + tourniquet group). In the eighth group, patients were intravenously injected with 1.0 g TXA was 10 min before incision and 1 g/20 mL normal saline TXA was injected into the joint after closure of the joint capsule with tourniquet used throughout the operation. The tourniquet was released after the incision was sutured and pressure bandaged (i.v.-combined topical TXA + tourniquet group).

#### Outcome measure

Total blood loss, the number of patients transfused, and hemoglobin (HB) 24 hs before and after operation were the main results, while DVT, PE, and operation time were the secondary results.

### Statistical analysis

Revman 5 software (version 5.3, Cochrane Collaboration, Oxford, UK) was used for evaluation and statistical analysis. The continuous data were calculated by weighted mean difference (WMDs) and 95% confidence interval (CI), while the binary classification data were calculated by risk ratio (OR) and 95% confidence interval (CI). Chi-square test and *I*^2^ test were used to test heterogeneity. If χ^2^ > 0.1 or *I*^2^ < 50%, then a fixed consequence model was used, and otherwise, a random effect model was used. The R 3.2.1 software was used to draw a network diagram.

The node-splitting method was used to evaluate the consistency between direct and indirect evidence, and the consistency or inconsistency model was selected based on the results. If the result of node segmentation showed *p* > 0.05, the consistency model was selected [19]. To assist in the interpretation of OR/WMDs, we calculated the probability of each intervention being the most effective treatment method following a Bayesian approach using probability values summarized as the surface under the cumulative ranking curve (SUCRA) [20, 21]. All computations were done using R (V.3.2.1) package gemtc (V.0.6), along with the Markov Chain Monte Carlo engine Open BUGS (V.3.4.0). All experimental data were processed and analyzed using the Statistic Package for Social Science (SPSS) 21.0 statistical software (IBM Corp. Armonk, NY, USA). Measurement data are presented as mean ± standard deviation. Comparisons among multiple groups were analyzed using one-way analysis of variance (ANOVA), followed by Tukey’s post hoc test. Enumeration data were analyzed by a chi-squared test. A value of *p* < 0.05 was considered to be statistically significant.

## Results

### The baseline characteristics of the included studies

**Fig. 1:**
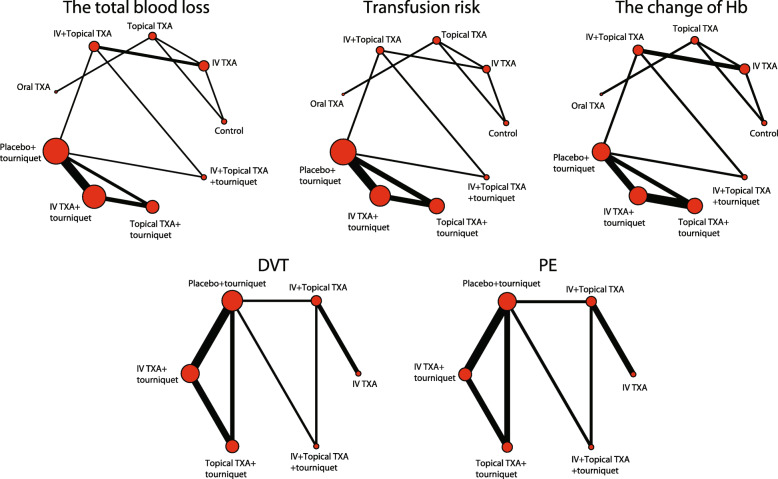
Evidence chart of the effect of different intervention methods on hemostasis in TKA

**Fig. 2:**
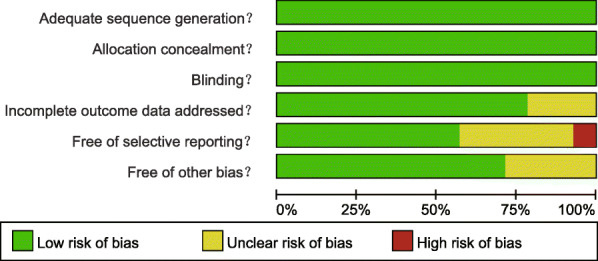
Cochrane system bias evaluation of included literature

**Fig. 3:**
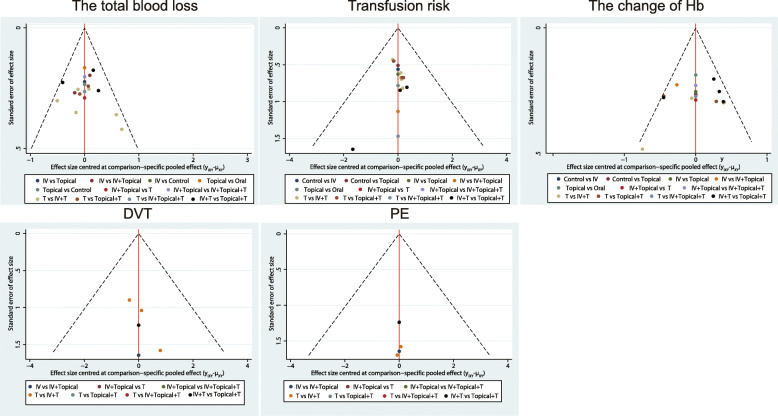
Funnel diagram of the effect of different intervention methods on hemostasis in TKA. Hb=hemoglobin; DVT=deep vein thrombosis; PE=pulmonary embolism; T=Tourniquets

In this study, there were 1498 related literature, 148 repetitive literature, 215 letters or reviews, and 108 non-human research literature. A total of 142 non-English literature were excluded. After the evaluation of the remaining 885 literature, 446 non-randomized controlled studies, 408 non-TKA literature, and 16 incomplete literature were excluded. Finally, 14 randomized controlled studies met the inclusion criteria used for this meta-analysis [5, 8, 22–34] (Fig. S1). A total of 1338 patients with osteoarthritis or rheumatoid arthritis and other diseases who had undergone corresponding operations, among whom the largest group had received i.v. injection of TXA drugs combined with a tourniquet (Fig. 1). The study was published from 1996 to 2018. Among them, 8 studies were from the European and American population, 6 from the Asian population, and 4 out of 14 studies were three armed studies and 10 were two armed studies. The baseline characteristics of the included literature are shown in Table 1, the Cochrane system bias evaluation is shown in Fig. 2, and the funnel plot is shown in Fig. 3.

**Table 1:** The baseline characteristics of included studies

Number	Author	Year	Country	Language	Disease	Number				Age(years)			Interventions			Interval, times, dose of TXA			Use of drainage tube	Outcomes
						Total	T1	T2	T3	T1	T2	T3	T1	T2	T3	T1	T2	T3		
1	Wang	2018	China	English	Osteoarthritis	147	74	73		65 ± 13.1	63.6 ± 11.5		E	C		2 g of TXA (4 tablets of 500 mg) by oral bolus appropriately 2 h before incision as a preoperative dose, a postoperative dose of 1 g was repeated 6 and 12 h after surgery, respectively	Received an intraarticular administration of 100 mL of saline solution containing a 3 g dose of TXA		No	Duration of surgery, the total blood loss, transfusion risk, Hb, ROM, DVT, PE
2	Huang	2017	China	English	Osteoarthritis	150	50	50	50	66.2 ± 8.3	65.1 ± 6.8	65.8 ± 6.3	I	D	F	Treated with a tourniquet multiple doses of intravenous TXA (20 mg/kg 5 to 10 min before the skin incision and 10 mg/kg 3, 6, 12, and 24 h later) along with 1 g of topical TXA	Multiple doses of intravenous TXA (20 mg/kg5 to 10 min before the skin incision and 10 mg/kg 3, 6, 12, and 24 h later) along with 1 g of topical TXA	Treated with the tourniquet only	Yes	ROM, Flexion contracture, Hb, the total blood loss, duration of surgery, transfusion risk, DVT, PE
3	Tzatzairis	2016	Greece	English	OSTEOARTHRITIS	120	40	40	40	68.58 ± 7.50	69.55 ± 6.61	69.10 ± 8.68	A	B	C	Did not receive TXA.	1 g (100 mg/mL) of TXA, in 100 mL normal saline, intravenously 10 min before incision	1 g (100 mg/mL) of TXA, in 100 mL normal saline was administered intra-articularly after joint capsule closure.	Yes	Blood loss, transfusion risk, Hb, duration of surgery
4	Nielsen	2016	Denmark	English	Osteoarthritis	60	30	30		65.5 ± 7.8	63.2 ± 8.6		B	D		Received 1 g of TXA administered intravenously only and 100 mL of saline solution administered intra-articularly	1 g of TXA administered intravenously during the induction of anesthesia and 3 g diluted in 100 mL of saline solution (0.9%) administered intra-articularly after closure of the capsule		No	Duration of surgery, the total blood loss, transfusion risk, Hb1d
5	Jain	2016	India	English	Osteoarthritis	119	60	59		70.0 ± 6.56	68.27 ± 8.66		B	D		Given IVTXA as a preoperative and postoperative dose given 3 and 6 h after surgery	topical TXA solution was applied intra-articularly about 5 min before closure of arthrotomyin addition IV doses		No	Duration of surgery, transfusion risk, the total blood loss, DVT, Hb
6	May	2016	USA	English	Osteoarthritis	131	69	62		65.0 ± 9.6	63.0 ± 10.6		G	H		1 g of TXA in 100 mL of normal saline (after anesthetic induction and before tourniquet inflation) and 1 dose of 50 mL of normal saline without TXA (administered after capsular closure)	2 g of TXA was prepared for the topical dose in 50 mL of saline, and 100 mL of normal saline without TXA		No	Duration of surgery, 0/1d Hb,3d total blood loss, PE, DVT
7	Drosos	2016	Greece	English	Osteoarthritis	90	30	30	30	71.77 ± 6.5	69.27 ± 7.21	71.1 ± 6.32	F	G	H	No TXA	Received 1 g TXA intravenously	1 g TXA in 30 ml normal saline (a solution of 40 ml) was applied topically	yes	Hb4d, the total blood loss, transfusion risk
8	Yang	2015	China	English	Osteoarthritis, traumatic arthritis or rheumatoid arthritis	80	40	40		69 ± 5	67 ± 6		F	H		20 mL of normal saline solution	Receive 500 mg of TXA in 20 mL of normal saline solution		No	Duration of surgery, Transfusion risk, Hb, DVT, PE
9	Keyhani	2015	Iran	English	Osteoarthritis	120	40	40	40	68.4 ± 10.4	67 ± 11.9	63.9 ± 9	G	H	F	Received 500 mg of TXA in 100 cc saline at the end of the surgery	Received an intra-articular dose of 3 g of TXA in 100 mL normal saline. Half of the solution was used to irrigate the joint before joint closure.	Did not receive TXA	Yes	Hb4d, the total blood loss, transfusion risk, DVT, PE
10	Patel	2014	USA	English	Osteoarthritis	89	42	47		64.9 ± 7.8	64.8 ± 9.7		G	H		Versus IV administration of 10 mg/kg, 10 min prior to tourniquet deflation.	Administered2.0gTXAin100mlofnormal saline directly into the surgical site and bathed in the solution, undisturbed for 2 min prior to tourniquet release		Yes	Hb, transfusion risk, DVT
11	Good	2003	Sweden	English	Osteoarthritis	51	24	27		72(50–84)	72(46–83)		F	G		Placebo 10 mg/kg i.v. just before tourniquet release and 3 h later	TXA 10 mg/kg i.v. just before tourniquet release and 3 h later		Yes	Blood loss, DVT, transfusion risk
12	Tanaka	2001	Japan	English	Osteoarthritis, rheumatoid arthritis	53	26	27		65(58–70)	65(59–69)		F	G		Saline twice, 10 min before surgery and on deflation of the tourniquet	10 mg/kg of TNA 10 min before surgery and again 10 min before deflation of the tourniquet	Saline 10 min before surgery and 20 mg/kg of TNA 10 min before deflation of the tourniquet	Yes	Hb1/2/4/7d, duration of surgery, transfusion risk, the total blood loss
13	Jansen	1999	Belgium	English	Osteoarthritis, rheumatoid arthritis	42	21	21		71.0(64–84)	70.7(62–80)		F	G		Normal saline of 15mgkg-1 was administered 30 min before surgery + every 8 h 3 days after surgery	TXA (15mgkg-1) was administered 30 min before surgery + every 8 h 3 days after surgery		Yes	Duration of surgery, the total blood loss, transfusion risk, DVT
14	BENON	1996	Sweden	English	Osteoarthritis, aseptic bone necrosis	86	43	43		76 ± 7	74 ± 7		G	F		IV 10 mg/kg TXA	A median time of 12 min (1 to 40) before deflation of the tourniquet, repeated after 3 h, 10 mg/kg placebo		Yes	Duration of surgery, the total blood loss, DVT, PE, ROM

*Notes*: *Hb* hemoglobin, *DVT* deep vein thrombosis, *PE* pulmonary embolism, *ROM* range of motion, A = placebo; B = IVTXA; C = topical TXA; D = IV combined topical TXA; E = oral TXA; F = placebo + tourniquets; G = IVTXA + tourniquets; H = topical TXA + tourniquets; I = IV-combined topical TXA + tourniquets; T1 = treatment 1; T2 = treatment 2; T3 = treatment 3

### Meta-analysis results

As shown in Table 2, in order to compare the hemostasis effect of 9 treatment measures on TKA, we made a direct matching comparison and found that in terms of blood loss, compared with the placebo group, the total blood loss of i.v. TXA group and topical TXA group was relatively less (WMD = −277.34, 95%CI = −431.85 to −122.83, WMD = −307.78, 95%CI = −460.92 to −154.64, respectively). Compared with the i.v. TXA group, the total blood loss of i.v.-combined topical TXA group reduced (WMD = −218.13, 95% CI = −282.59–153.68). Compared with i.v.-combined topical group, there was higher total blood loss in the placebo + tourniquet group and i.v.-combined topical TXA + tourniquet group (WMD = 956.60, 95%CI = 829.31–1083.89, (WMD = 106.80, 95%CI = 13.04–200.56, respectively). Compared with the placebo + tourniquet group, the total blood loss in the i.v. TXA + tourniquet group, the topical TXA + tourniquet group and the i.v.-combined topical TXA + tourniquet group was relatively less (WMD = −156.09, 95%CI = −179.41 to −132.78, WMD = −79.20, 95%CI = −106.34 to −52.05, WMD = −849.80, 95%CI = −987.51 to −712.09, respectively). In terms of blood transfusion risk, compared with the placebo group, the i.v. TXA group and the topical TXA group had a relatively low blood transfusion risk (or = 0.23, 95% CI = 0.08–0.74, or = 0.35, 95% CI = 0.13–1, perspective). Compared with the placebo + tourniquet group, the i.v. TXA + tourniquet group, the topical TXA + tourniquet group, and the i.v.-combined topical TXA + tourniquet group had a relatively low blood transfusion risk (OR = 0.09, 95%CI = 0.04–0.17, OR = 0.19, 95%CI = 0.10-0.37, OR = 0.05, 95%CI = 0.00–0.88, respectively). In terms of the change value of hemoglobin, compared with the placebo group, the reduction value of hemoglobin in the i.v.-combined topical TXA group was relatively low (WMD = 1.17, 95% CI = 0.54–1.80, WMD = 1.42, 95% CI = 0.78–2.06, respectively), compared with the i.v.-combined topical TXA group, the reduction value of hemoglobin in the i.v.-combined topical TXA group was relatively low (WMD = 0.67, 95% CI = 0.48–0.86). Compared with the i.v.-combined topical TXA group, there was more reduction of the hemoglobin value in the placebo + tourniquet group and the i.v.-combined topical TXA + tourniquet group (WMD = −1.61, 95%CI = −1.90 to −1.32, WMD = −0.20, 95%CI = −0.38 to −0.02, respectively). Compared with the placebo + tourniquet group, there was less reduction in the hemoglobin value in the i.v. TXA + tourniquet group, topical TXA + tourniquet group, and the i.v.-combined topical TXA + tourniquet group (WMD = 1.41, 95%CI = 1.11–1.71, respectively). Compared with the i.v. TXA + tourniquet group, the hemoglobin reduction in the topical TXA + tourniquet group was relatively low (WMD = 0.57, 95%CI = 0.31–0.84) (Table 2).

**Table 2:** Estimated OR/WMD and 95% CI from pairwise meta-analysis in terms of Blood loss, Transfusion risk, Change of Hb, DVT and PE

Included studies	Comparisons	Pairwise meta-analysis		
		WMD(95%CI)	***I***^**2**^	***P***_***h***_
**The total blood loss** (PMID:29544472;PMID:29257010;PMID:27267228;PMID:27194493;PMID:26507526;PMID:27259391;PMID:27222617;PMID:26894222;PMID:12697586;PMID:11476309;PMID:10673876; PMID:8636182)				
1 study	IV vs. control	**−277.34 (−431.85–122.83)**	NA	NA
1 study	Topical vs. control	**−307.78 (−460.92–154.64)**	NA	NA
1 study	Topical vs. IV	**−**30.44 (**−**163.81–102.93)	NA	NA
2 studies	IV + topical vs. IV	**−218.13 (−282.59–153.68)**	46.8%	0.17
1 study	Oral vs. topical	**−**83.60 (**−**203.84–36.64)	NA	NA
1 study	T VS. IV + topical	**956.60 (829.31–1083.89)**	NA	NA
6 studies	IV + T vs. T	**−156.09 (−179.41–132.78)**	98.1%	<0.01
2 studies	Topical+T vs. T	**−79.20 (−106.34–52.05)**	87.6%	<0.01
3 studies	Topical+T vs. IV + T	10.90 (**−**7.95–29.72)	62.5%	0.07
1 study	IV + topical+T vs. IV + topical	**106.80 (13.04–200.56)**	NA	NA
1 study	IV + topical+T vs. T	**−849.80(−987.51–712.09)**	NA	NA
**Transfusion risk** (PMID:29544472;PMID:29257010;PMID:27267228;PMID:26507526;PMID:27222617;PMID:24816760;PMID:26894222;PMID:24768543;PMID:12697586;PMID:11476309;PMID:10673876)				
1 study	IV vs. control	**0.23 (0.08–0.74)**	NA	NA
1 study	Topical vs. control	**0.35 (0.13–1.00)**	NA	NA
1 study	Topical vs. IV	1.48 (0.43–5.14)	NA	NA
1 study	IV + topical vs. IV	0.24 (0.03–2.23)	NA	NA
1 study	Oral vs. topical	0.73 (0.16–3.38)	NA	NA
1 study	T VS. IV + topical	20.20 (1.13–360.28)	NA	NA
5 studies	IV + T vs. T	**0.09 (0.04–0.17)**	0%	0.77
3 studies	Topical+T vs. T	**0.19 (0.10–0.37)**	46.2%	0.16
3 studies	Topical+T vs. IV + T	0.99 (0.34–2.85)	0%	0.76
1 study	IV + Topical+T vs. T	**0.05 (0.00–0.88)**	NA	NA
**The change of Hb** (PMID:29544472;PMID:29257010;PMID:27267228;PMID:27194493;PMID:26507526;PMID:27259391;PMID:27222617;PMID:26894222;PMID:24768543;PMID:11476309)				
1 study	IV vs. control	**1.17 (0.54–1.80)**	NA	NA
1 study	Topical vs. control	**1.42 (0.78–2.06)**	NA	NA
1 study	Topical vs. IV	0.25 (**−**0.32–0.82)	NA	NA
2 studies	IV + topical vs. IV	**0.67 (0.48–0.86)**	0%	0.81
1 study	Oral vs. topical	0.20 (**−**0.13–0.53)	NA	NA
1 study	T VS. IV + topical	**−1.61 (−1.90–1.32)**	NA	NA
3 studies	IV + T vs. T	**2.50 (2.09–2.90)**	94.8%	<0.01
2 studies	Topical+T vs. T	**1.42 (0.84–2.00)**	5.4%	0.30
4 studies	Topical+T vs. IV + T	**0.57 (0.31–0.84)**	92.0%	<0.01
1 study	IV + topical+T vs. IV + topical	**−0.20 (−0.38–0.02)**	NA	NA
1 study	IV + topical+T vs. T	**1.41 (1.11–1.71)**	NA	NA
**DVT** (PMID:29257010;PMID:27194493;PMID:26507526;PMID:27259391;PMID:24816760;PMID:26894222;PMID:24768543;PMID:12697586;PMID:10673876; PMID:8636182)				
1 study	IV + topical vs. IV	0.33 (0.01–8.35)	NA	NA
3 studies	IV + T vs.T	0.80 (0.24–2.63)	0%	0.50
1 study	Topical+T vs. IV + T	0.55 (0.05–6.21)	NA	NA
**PE** (PMID:29257010;PMID:27194493;PMID:26507526;PMID:27259391;PMID:24816760;PMID:26894222;PMID:10673876;PMID:8636182)				
1 study	IV + topical vs. IV	0.33 (0.01–8.35)	NA	NA
2 studies	IV + T vs. T	0.22 (0.24–2.14)	0%	0.83
1 study	Topical+T vs. IV + T	0.55 (0.05–6.21)	NA	NA

*Notes*: *WMD* weighted mean difference, *95%CI* 95% confidence intervals, *Hb* hemoglobin, *DVT* deep vein thrombosis, *PE* pulmonary embolism, Control = placebo; IV=IV TXA; topical = topical TXA; IV + topical = IV-combined topical TXA; Oral = oral TXA; T = placebo+tourniquets; IV + T = IV TXA + tourniquets; Topical+T = topical TXA + tourniquets; IV + topical+T = IV-combined topical TXA + tourniquets

**Fig. 4:**
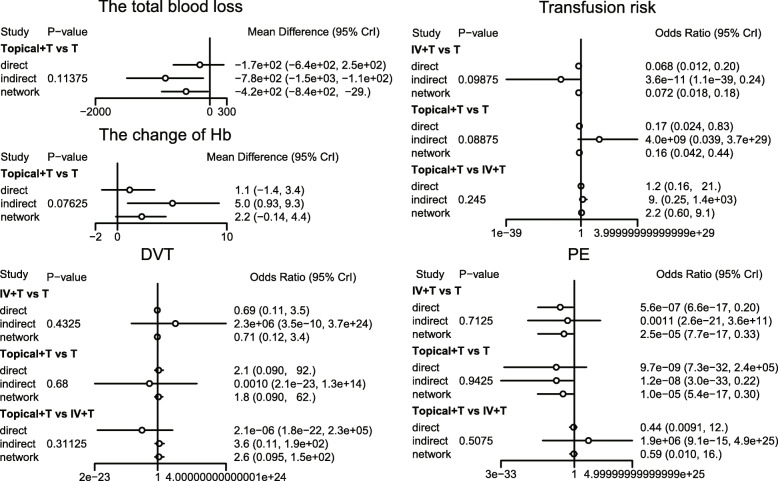
Node cutting diagram of the influence of different intervention methods on hemostasis in TKA

The results of the five outcome indicators, including total blood loss, risk of blood transfusion, hemoglobin change, DVT, and PE were analyzed by the method of node segmentation. It was found that the direct evidence and indirect evidence of all outcome indicators were consistent, and the consistency model should be used (all *p* > 0.05) (Fig. 4).

**Fig. 5:**
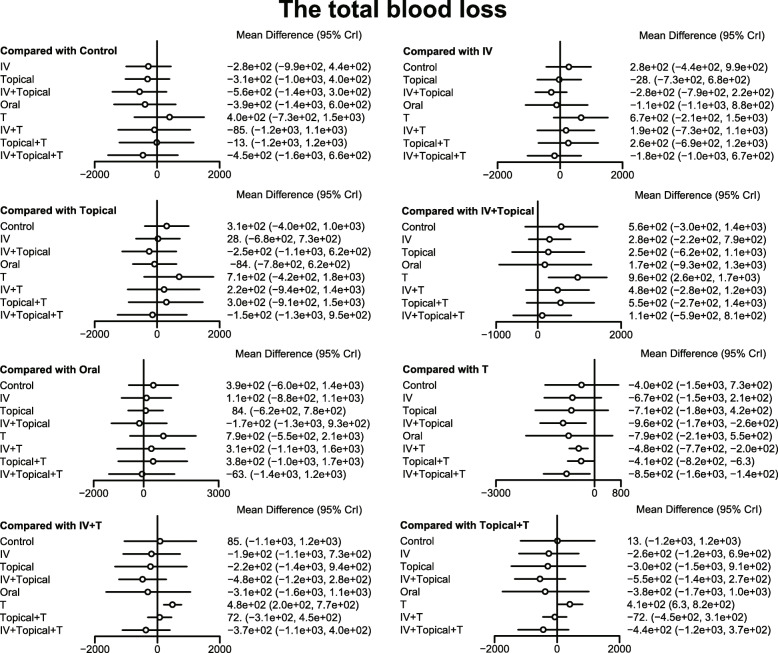
Forest map of the effect of total blood loss on hemostasis in TKA

**Fig. 6:**
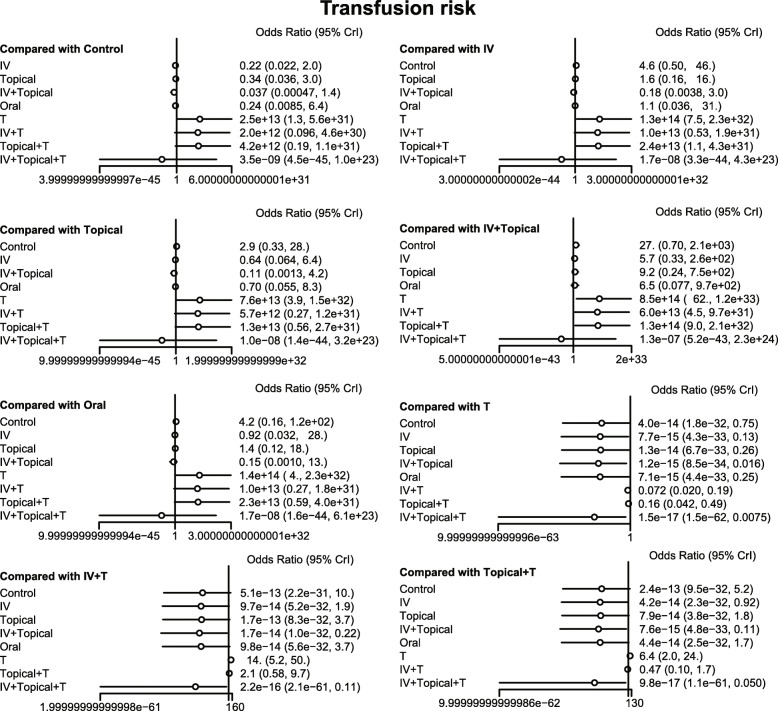
Forest map of the effect of transfusion risk on hemostasis in TKA

**Fig. 7:**
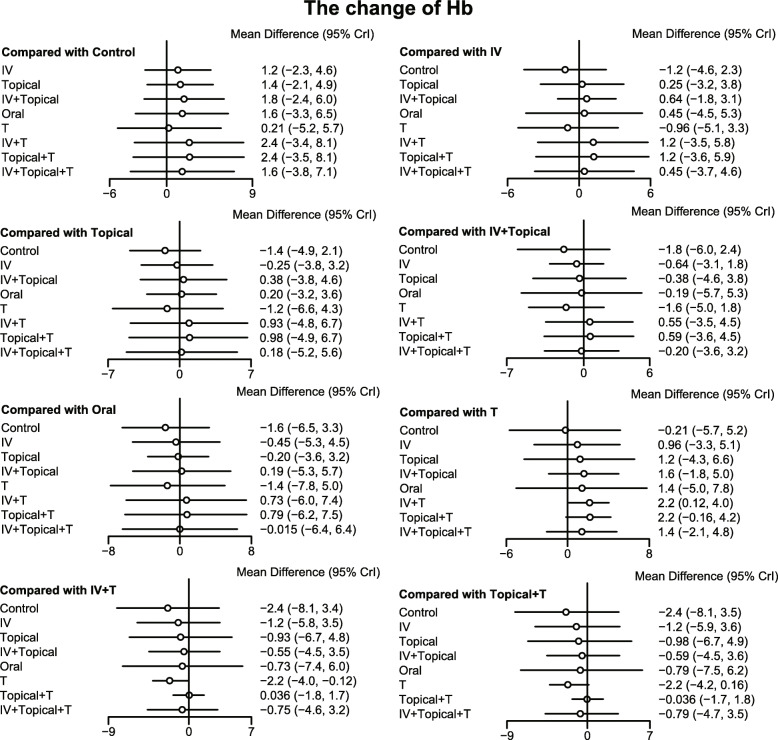
Forest map of the effect of change value of hemoglobin on hemostasis in TKA

**Fig. 8:**
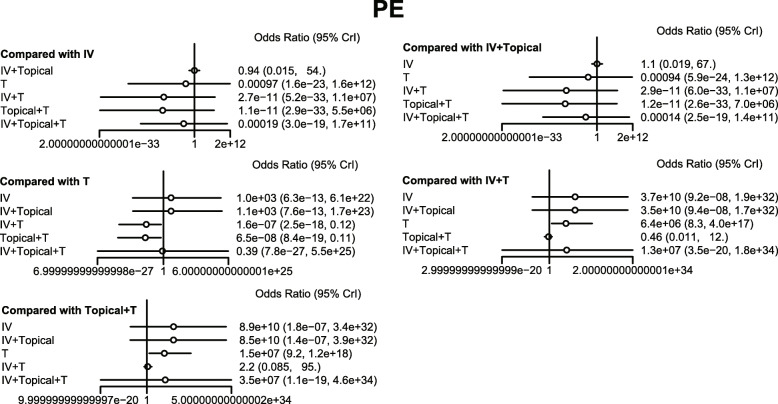
Forest map of the effect of PE on hemostasis in TKA

The results of the meta-analysis showed that in comparison to the placebo + tourniquet group, the total blood loss decreased in i.v.-combined topical TXA group, i.v. TXA + tourniquet group, topical TXA + tourniquet group, combined i.v. plus topical TXA + tourniquet group. Thus, the hemostasis effect was relatively good in these four groups. Compared with the placebo + tourniquets group, the risk for blood transfusion was lower in the i.v. TXA + tourniquet group, the topical TXA + tourniquet group, and the i.v.-combined topical TXA + tourniquet group. Compared with i.v. TXA + tourniquet and topical TXA + tourniquet groups, blood transfusion risk was reduced in the i.v.-combined topical TXA + tourniquets group (OR = 0.00, 95%CI = 0.00–0.31; OR = 0.00, 95%CI = 0.00–0.10, respectively). Compared with the placebo + tourniquet group, the i.v. TXA + tourniquet group had a relatively low hemoglobin reduction (WMD = 2.15, 95%CI = 0.17–4.06). Compared with the placebo + tourniquet group, the incidence of PE was lower in the i.v. TXA + tourniquet group and the topical TXA + tourniquet group (OR = 0.00, 95%CI = 0.00–0.21). However, in terms of DVT incidence, there was no significant difference in the effect of each intervention in TKA (Figs. 5, 6, 7, 8 and Table 3).

**Table 3:** OR/WMD and 95% confidence intervals of nine treatment modalities of five end point outcomes

OR/WMD(95%CI)								
**The total blood loss**								
**Control**	−279.54 (−992.45, 434.18)	−308.96 (−1024.88, 426.78)	−562.67 (−1435.57, 299.48)	−394.27 (−1396.04, 632.56)	386.91 (−705.21, 1525.93)	−96.32 (−1230.91, 1077.73)	−28.850 (−1195.33, 1191.28)	−469.22 (−1572.29, 634.08)
279.54 (−434.18, 992.45)	**IV**	−28.22 (−734.22, 662.79)	−282.77 (−780.12, 207.94)	−115.28 (−1079.06, 870.79)	671.39 (−195.64, 1524.22)	186.49 (−707.88, 1110.99)	252.16 (−687.87, 1212.63)	−179.78 (−1068.64, 682.97)
308.96 (−426.78, 1024.88)	28.22 (−662.79, 734.22)	Topical	−245.88 (−1105.28, 602.53)	−79.97 (−777.45, 615.71)	697.78 (−380.83, 1813.94)	214.28 (−906.59, 1355.67)	283.58 (−876.70, 1470.69)	−147.79 (−1272.04, 918.77)
562.67 (−299.48, 1435.57)	282.77 (−207.94, 780.12)	245.88 (−602.53, 1105.28)	IV + Topical	165.30 (−912.01, 1256.91)	**954.88 (266.02, 1654.51)**	471.25 (−273.91, 1238.80)	540.05 (−273.34, 1359.22)	107.35 (−602.64, 788.28)
394.27 (−632.56, 1396.04)	115.28 (−870.79, 1079.06)	79.97 (−615.71, 777.45)	−165.30 (−1256.91, 912.01)	Oral	784.26 (−507.66, 2069.12)	304.93 (−1045.00, 1636.16)	373.44 (−989.60, 1741.73)	−62.52 (−1380.86, 1215.63)
−386.91 (−1525.93, 705.21)	−671.39 (−1524.22, 195.64)	−697.78 (−1813.94, 380.83)	−**954.88 (**−**1654.51,** −**266.02)**	−784.26 (−2069.12, 507.66)	T	−**482.09 (**−**768.30,** −**195.69)**	−**410.97 (**−**828.87,** −**3.30)**	−**850.19 (**−**1568.81,** −**151.31)**
96.32 (−1077.73, 1230.91)	−186.49 (−1110.99, 707.88)	−214.28 (−1355.67, 906.59)	−471.25 (−1238.80, 273.91)	−304.93 (−1636.16, 1045.00)	**482.09 (195.69, 768.30)**	IV + T	70.28 (−314.39, 455.56)	−372.67 (−1148.75, 370.06)
28.85 (−1191.28, 1195.33)	−252.16 (−1212.63, 687.87)	−283.58 (−1470.69, 876.70)	−540.05 (−1359.22, 273.34)	−373.44 (−1741.73, 989.60)	**410.97 (3.30, 828.87)**	−70.28 (−455.56, 314.39)	Topical+T	−441.48 (−1268.95, 369.63)
469.22 (−634.08, 1572.29)	179.78 (−682.97, 1068.64)	147.79 (−918.77, 1272.04)	−107.35 (−788.28, 602.64)	62.52 (−1215.63, 1380.86)	**850.19 (151.31, 1568.81)**	372.67 (−370.06, 1148.75)	441.48 (−369.63, 1268.95)	IV + Topical+T
**Transfusion**								
**Control**	0.23 (0.02, 2.03)	0.36 (0.04, 2.88)	0.04 (0.00, 1.45)	0.27 (0.01, 5.87)	—>100 (31.61, —>100)	—>100 (3.78,—>100)	—>100 (6.98, —>100)	0.00 (0.00,—>100 )
4.40 (0.49, 43.87)	**IV**	1.51 (0.17, 16.26)	0.17 (0.00, 2.92)	1.07 (0.04, 28.47)	—>100 (—>100, —>100)	—>100 (23.94,—>100)	—>100 (41.90, —>100)	0.00 (0.00,—>100 )
2.80 (0.35, 25.73)	0.66 (0.06, 5.82)	**Topical**	0.11 (0.00, 3.93)	0.75 (0.06, 7.87)	—>100 (87.19, —>100)	—>100 (9.74,—>100)	—>100 (16.69, —>100)	0.00 (0.00,—>100 )
26.89 (0.69, 5597.56)	5.85 (0.34, 769.88)	9.17 (0.25, 1741.44)	**IV + Topical**	6.41 (0.08, 1831.07)	—>100 (—>100, —>100)	—>100 (281.87,—>100)	—>100 (500.86, —>100)	0.00 (0.00,—>100 )
3.74 (0.17, 107.08)	0.94 (0.04, 24.79)	1.33 (0.13, 17.90)	0.16 (0.00, 12.87)	**Oral**	—>100 (75.55, —>100)	—>100 (6.85, —>100)	—>100 (12.70, —>100)	0.00 (0.00,—>100 )
0.00 (0.00, 0.03)	0.00 (0.00, 0.01)	0.00 (0.00, 0.01)	0.00 (0.00, 0.00)	0.00 (0.00, 0.01)	**T**	**0.07 (0.02, 0.18)**	**0.16 (0.04, 0.48)**	**0.00 (0.00, 0.02)**
0.00 (0.00, 0.26)	0.00 (0.00, 0.04)	0.00 (0.00, 0.10)	0.00 (0.00, 0.00)	0.00 (0.00, 0.15)	14.15 (5.45, 50.16)	**IV + T**	2.24 (0.62, 9.67)	**0.00 (0.00, 0.31)**
0.00 (0.00, 0.14)	0.00 (0.00, 0.02)	0.00 (0.00, 0.06)	0.00 (0.00, 0.00)	0.00 (0.00, 0.08)	**6.31 (2.10, 22.26)**	0.45 (0.10, 1.63)	**Topical + T**	**0.00 (0.00, 0.10)**
—>100 (0.00,—>100)	—>100 (0.00,—>100)	—>100 (0.00, —>100)	—>100 (0.00,—>100)	—>100 (0.00,—>100)	**—>100 (52.32, —>100)**	**—>100 (3.27, —>100)**	**—>100 (9.66, —>100)**	**IV + Topical + T**
**Change of Hb**								
**Control**	1.19 (−2.48, 4.69)	1.43 (−2.08, 4.96)	1.82 (−2.59, 6.14)	1.66 (−3.16, 6.52)	0.19 (−5.43, 5.95)	2.32 (−3.71, 8.35)	2.39 (−3.86, 8.41)	1.65 (−4.10, 7.16)
−1.19 (−4.69, 2.48)	**IV**	0.27 (−3.29, 3.84)	0.66 (−1.81, 3.21)	0.46 (−4.42, 5.67)	−0.97 (−5.28, 3.45)	1.14 (−3.52, 6.03)	1.21 (−3.70, 6.13)	0.45 (−3.79, 4.84)
−1.43 (−4.96, 2.08)	−0.27 (−3.84, 3.29)	**Topical**	0.38 (−4.04, 4.76)	0.21 (−3.14, 3.66)	−1.23 (−6.82, 4.38)	0.92 (−5.10, 6.92)	0.97 (−5.24, 6.89)	0.21 (−5.44, 5.76)
−1.82 (−6.14, 2.59)	−0.66 (−3.21, 1.81)	−0.38 (−4.76, 4.04)	**IV + Topical**	−0.16 (−5.73, 5.46)	−1.61 (−5.18, 1.86)	0.52 (−3.55, 4.49)	0.58 (−3.76, 4.61)	−0.20 (−3.69, 3.26)
−1.66 (−6.52, 3.16)	−0.46 (−5.67, 4.42)	−0.21 (−3.66, 3.14)	0.16 (−5.46, 5.73)	**Oral**	−1.42 (-8.13, 5.12)	0.70 (-6.26, 7.59)	0.78 (−6.51, 7.58)	−0.02 (−6.84, 6.43)
−0.19 (−5.95, 5.43)	0.97 (−3.45, 5.28)	1.23 (−4.38, 6.82)	1.61 (−1.86, 5.18)	1.42 (−5.12, 8.13)	T	**2.15 (0.17, 4.06)**	2.18 (−0.22, 4.33)	1.42 (−2.07, 4.87)
−2.32 (−8.35, 3.71)	−1.14 (−6.03, 3.52)	−0.92 (−6.92, 5.10)	-0.52 (−4.49, 3.55)	−0.70 (−7.59, 6.26)	−**2.15 (**−**4.06,** −**0.17)**	**IV + T**	0.03 (−1.78, 1.73)	−0.73 (−4.67, 3.27)
−2.39 (−8.41, 3.86)	−1.21 (−6.13, 3.70)	−0.97 (−6.89, 5.24)	−0.58 (−4.61, 3.76)	−0.78 (−7.58, 6.51)	−2.18 (−4.33, 0.22)	−.03 (−1.73, 1.78)	**Topical + T**	−0.76 (−4.73, 3.45)
−1.65 (−7.16, 4.10)	−0.45 (−4.84, 3.79)	−0.21 (−5.76, 5.44)	0.20 (−3.26, 3.69)	0.02 (−6.43, 6.84)	−1.42 (−4.87, 2.07)	0.73 (−3.27, 4.67)	0.76 (−3.45, 4.73)	
**DVT**								
**IV**	0.00 (0.00, 3.45)	0.00 (0.00,—>100)	0.00 (0.00,—>100)	0.00 (0.00,—>100)	0.00 (0.00,—>100)			
—>100 (0.29, —>100 )	**IV + Topical**	0.07 (0.00, —>100)	0.04 (0.00, —>100 )	0.16 (0.00, —>100 )	0.01 (0.00, —>100 )			
—>100 (0.00,—>100)	14.38 (0.00, —>100 )	T	0.72 (0.11, 3.67)	2.03 (0.09, 47.51)	7.54 (0.00,—>100 )			
—>100 (0.00,—>100)	24.63 (0.00, —>100 )	1.39 (0.27, 9.28)	**IV + T**	2.75 (0.09, 116.04)	6.40 (0.00, —>100 )			
—>100 (0.00,—>100)	6.18 (0.00, —>100 )	0.49 (0.02, 11.22)	0.36 (0.01, 11.57)	**Topical + T**	1.79 (0.00, —>100 )			
—>100 (0.00,—>100)	93.11 (0.00, —>100 )	0.13 (0.00,—>100 )	0.16 (0.00, —>100 )	0.56 (0.00,—>100 )	**IV + Topical + T**			
**PE**								
**IV**	1.06 (0.01, 96.80)	0.09 (0.00, —>100)	0.00 (0.00,—>100)	0.00 (0.00,—>100)	0.01 (0.00, —>100)			
0.94 (0.01, 67.85)	**IV + Topical**	0.08 (0.00, —>100)	0.00 (0.00,—>100)	0.00 (0.00,—>100)	0.01 (0.00,—>100)			
10.78 (0.00, —>100)	12.88 (0.00, —>100)	T	**0.00 (0.00, 0.26)**	**0.00 (0.00, 0.21)**	0.01 (0.00, —>100 )			
—>100 (0.00,—>100)	—>100 (0.00,—>100)	**—>100 (3.84,—>100)**	**IV + T**	0.47 (0.01, 13.69)	—>100 (0.00,—>100)			
—>100 (0.00,—>100)	—>100 (0.00,—>100)	**—>100 (4.82, —>100)**	2.13 (0.07, 81.42)	**Topical + T**	—>100 (0.00,—>100)			
—>100 (0.00,—>100)	—>100 (0.00,—>100)	71.96 (0.00,—>100)	—>100 (0.00,—>100)	0.00 (0.00,—>100 )	**IV + Topical + T**			

*Notes*: *Hb* hemoglobin, *DVT* deep vein thrombosis, *PE* pulmonary embolism; Control = Placebo; IV=IV TXA; Topical = topical TXA; IV + Topical = IV combined topical TXA; Oral = Oral TXA; T = Placebo+tourniquets; IV + T = IV TXA + tourniquets; Topical+T = topical TXA + tourniquets; IV + Topical+T = IV-combined topical TXA + tourniquets; .—>100 = Far more than 100

As shown in Table 4, the cumulative ranking probability SUCRA value of different interventions on hemostasis in TKA, in terms of total blood loss, blood transfusion risk, and hemoglobin change, the cumulative sorting probability of placebo + tourniquet group was higher than that of the other eight groups (94.22%, 100%, 80.26%, respectively), suggesting that the hemostasis effect in the placebo + tourniquet group is relatively poor in TKA. The total blood loss (26.56%) and the risk of blood transfusion (24.33%) in the i.v.-combined topical TXA group were lower than in the other eight groups, indicating a superior hemostasis effect. The cumulative sorting probability of hemoglobin reduction (37.46%) in the topical TXA + tourniquets group was lower than that in the other eight groups

**Table 4:** Cumulative ranking probability of TKR main outcome indicators by different treatment measures (surface under the cumulative ranking curves, SUCRA)

	The total blood loss	Transfusion	Change of Hb
Control	2(87.67%)	4(58.56%)	2(79.84%)
IV TXA	5(54.89%)	7(38.22%)	3(60.80%)
Topical TXA	3(58.00%)	5(43.44%)	4(54.22%)
IV+topical TXA	9(24.44%)	9(24.33%)	7(46.14%)
Oral TXA	6(50.22%)	6(38.67%)	6(51.29%)
Placebo+tourniquet	1(90.44%)	1(100.00%)	1(80.26%)
IV TXA+tourniquet	7(49.44%)	3(78.78%)	8(37.87%)
Topical TXA+tourniquet	4(56.33%)	2(86.89%)	9(37.46%)
IV+topical TXA+tourniquet	8(29.22%)	8(28.56%)	5(52.13%)

*Hb* hemoglobin

### Retrospective analysis of clinical research results

As shown in Table 5, different treatment methods of TXA and tourniquet were significantly related to the total blood loss of TKA, the number of people who needed a blood transfusion, and the change of hemoglobin before and after the operation (all *p* < 0.05), there was no significant correlation with gender, age, knee joint activity, operation time, and postoperative complications (all *p* > 0.05). Among them, the indexes of i.v.-combined topical TXA group were better than the other seven groups.

**Table 5:** Baseline characteristics and intraoperative and postoperative outcomes

Group	IV TXA	TopicalTXA	IV-combined topical TXA	OralTXA	Placebo + tourniquets	IV TXA + tourniquets	Topical TXA + tourniquets	IV-combined topical \TXA + tourniquets	*P* value
Number	30	30	30	30	30	30	30	30	
Age(y)	67.77 ± 5.39	67.37 ± 5.49	66.73 ± 6.15	66.3 ± 4.89	65.23 ± 6.28	65.73 ± 5.99	67.13 ± 5.56	65.43 ± 4.78	*0.532*
Gender(female/male)	16/14	20/10	21/9	15/15	18/12	16/14	22/8	21/9	0.394
BMI(kg/m2)	30.05 ± 3.02	30.43 ± 4.28	30.72 ± 2.93	30.54 ± 3.09	29.49 ± 3.87	29.62 ± 3.37	31.41 ± 3.62	31.33 ± 4.11	0.307
Preop.ROM(°)	99.87 ± 8.33	100.01 ± 7.83	103.45 ± 5.35	101.53 ± 6.28	102.08 ± 6.72	99.37 ± 5.04	100.88 ± 6.98	102.41 ± 7.73	0.238
Surgical duration(min)	67.60 ± 12.39	64.93 ± 10.79	68.13 ± 8.97	66.03 ± 12.17	66.07 ± 9.75	64.00 ± 8.53	63.37 ± 12.18	70.10 ± 11.18	0.256
Total blood loss(mL)	1268.84 ± 342.15	1164.27 ± 304.19	653.79 ± 246.20	781.26 ± 213.79	1584.33 ± 359.21	1049.12 ± 266.72	1002.48 ± 316.29	739.15 ± 226.37	<0.001
Transfusion	9	5	1	7	12	3	5	4	0.009*
Preop.Hb(g/dL)	13.87 ± 0.66	13.44 ± 1.23	13.92 ± 1.25	13.89 ± 0.95	13.62 ± 1.02	13.41 ± 1.07	13.99 ± 1.09	13.47 ± 0.72	0.117
24-h Hb(g/dL)	11.82 ± 1.04	11.49 ± 1.30	12.09 ± 1.40	12.74 ± 0.94	10.37 ± 1.26	11.48 ± 1.35	11.94 ± 1.31	12.11 ± 0.95	<0.001*
Hb change(g/dL)	2.02 ± 0.84	1.95 ± 0.57	1.83 ± 0.61	1.14 ± 0.39	3.22 ± 1.06	1.93 ± 0.75	2.09 ± 0.61	1.36 ± 0.55	<0.001*
DVT	1	0	0	0	0	1	0	0	0.541
PE	0	0	0	0	0	0	1	0	0.432

*Notes*: *Preop.ROM* preoperative range of motion, *Preop.Hb* preoperative hemoglobin. Measurement data are presented as mean ± standard deviation. Comparisons among multiple groups were analyzed using ANOVA, followed by Tukey’s post hoc test. Enumeration data were analyzed by chi-square test. *p < 0.05

## Discussion

TKA is a complex surgery that often brings life-transforming improvements in function, but perioperative complications still arise, notably in relation to excessive intra- and post-operative blood loss [12]. There is general acceptance that the application of tourniquet reduces intraoperative blood loss after TKA [8]. Moreover, various dosing regimens used for TXA have proven effective, including intra-articular (IA) and oral forms, likewise i.v. TXA alone or in conjunction with topical TXA. However, there is meager scientific evidence for the benefits of combining i.v. and topical TXA in the course of TKA [35]. Therefore, we conducted a network meta-analysis to assess hemostatic effects of nine variant treatment methods following TKA in this study. Based on the network analysis and direct and indirect comparisons, the results of our retrospective clinical study indicated that i.v.-combined topical TXA was superior to the other eight variants with respect to low total blood loss and low risk of need for blood transfusion.

Initially, pairwise meta-analysis revealed that compared with the i.v. TXA, placebo + tourniquet, and i.v.-combined topical TXA + tourniquet groups, the i.v.-combined topical TXA group displayed less total blood loss and less reduction in hemoglobin value. Compared with the placebo and placebo + tourniquet, i.v. TXA groups, the topical TXA, the i.v.-combined topical TXA, i.v. TXA + tourniquet, topical TXA + tourniquet, and i.v.-combined topical TXA + tourniquet groups revealed decreased blood transfusion risk and hemoglobin reduction value. Tourniquet application in TKA brings benefits of reduced intraoperative blood loss and operation time, but without significantly decreasing the need for blood transfusion or the rate of DVT in TKA [7]. However, using a tourniquet brings a certain risk of muscle damage, severe thigh pain, delayed rehabilitation, reduced patient satisfaction, and even paralysis due to nerve damage. Thus, some have speculated that TKA without tourniquet can avoid those adverse effects while promoting postoperative recovery [6]. Moreover, recent RCTs have proved that the routine use of TXA in TKA reduces blood loss and transfusion rates [36]. A prior study has evaluated the risks and benefits of tourniquet use compared with i.v. and topical administration of TXA in the setting of primary TKA, which revealed that i.v. and topical TXA without a tourniquet had less hidden blood loss, a lower incidence of postoperative knee swelling, less postoperative knee pain, lower levels of inflammatory biomarkers, and better early knee function along with greater ratings of patient satisfaction compared with those treated with a tourniquet [22]. Santias et al. have demonstrated that topical administration of TXA without tourniquet reduces blood loss and improves postoperative blood chemistries in patients receiving TKA without increasing the risk of thromboembolic complications [15]. In a study of 640 patients treated with either i.v. or topical TXA, greater blood loss was seen in the patients who received topical TXA, whereas i.v. administration achieved an immediate and desirable effect, especially in the absence of a tourniquet [13].

Additionally, NMA combined with the retrospective clinical study further validated that i.v.-combined topical TXA provides a low total blood loss without increasing blood transfusion risk. Additionally, this combined treatment had the lowest SUCRA values in terms of total blood loss (24.44%) and blood transfusion risk (24.33%), whereas topical TXA + tourniquet had the lowest SUCRA values in terms of hemoglobin reduction value (37.46%), which also proved that i.v.-combined topical TXA had the best hemostatic effects of tourniquet + TXA in TKA. Similarly, a recent study has displayed that, compared with either i.v. TXA or topical administration TXA alone, the combined administrated TXA can decrease the total blood loss, and the difference is statistically significant, whereas the pooled results indicated that combined topical with i.v. TXA decreased the need for transfusion [16]. Jain et al. have also concluded that combined i.v. with topical TXA provided better results than did i.v. alone with respect to mean calculated total blood loss, blood transfusion rate, hemoglobin drop, and the absence of significant effect on DVT [25]. These findings support that i.v.-combined topical TXA achieves better hemostatic effect following TKA.

However, there was a limitation in this study. The differences in the sample sizes of the nine variant interventions and the number of studies included in the direct matching comparison between various interventions have some impact on the research results. In this study, the hemostatic effect of TXA combined with a tourniquet on TKA was compared comprehensively from the perspectives of NMA and clinical research, which revealed important differences in the hemostatic outcomes of the nine variant intervention measures, notwithstanding statistical issues related to sample sizes in the literature.

## Conclusion

In conclusion, i.v.-combined topical TXA contributes to lower total blood loss and blood transfusion risk in TKA patients. The i.v.-combined topical TXA proved to be superior to the other eight variant regiments, which calls for its adoption as standard care in TKA.

## Supplementary information

**Additional file 1: Fig. S1.** Flow chart of literature screening

## Data Availability

The datasets generated/analyzed during the current study are available.

## Abbreviations

TKA: Total knee arthroplasty
TXA: Tranexamic acid
RCTs: Randomized controlled trials
DVT: Deep vein thrombosis
i.v.: Intravenous
PE: Pulmonary embolism
CI: Confidence interval
SUCRA: Surface under the cumulative ranking curve
IA: Intra-articular
